# Correlation of Heart Rate Variability as a Measure of Autonomic Dysregulation in Stable Chronic Obstructive Pulmonary Disease

**DOI:** 10.7759/cureus.107493

**Published:** 2026-04-21

**Authors:** Dinakaran Umashankar, Karthikeyan Ramaraju, Anupama Murthy, Nagashree R

**Affiliations:** 1 Internal Medicine, Trinity Health Oakland, Pontiac, USA; 2 Respiratory Medicine, PSG Institute of Medical Sciences and Research/PSG Hospitals, Coimbatore, IND; 3 Respiratory Medicine, PSG Institute of Medical Sciences and Research, Coimbatore, IND; 4 Physiology, PSG Institute of Medical Sciences and Research, Coimbatore, IND

**Keywords:** autonomic nervous system, cardiovascular risk, chronic obstruction pulmonary disease, heart rate variability, lung physiology

## Abstract

Background: Chronic obstructive pulmonary disease (COPD) affects not only the lungs but also the cardiovascular system and other organs, contributing to high morbidity and mortality. Recent studies suggest that patients with COPD exhibit autonomic dysfunction, which may increase the risk of cardiovascular mortality. Therefore, early detection and close monitoring of autonomic dysfunction in COPD patients are essential.

Methods: Patients were recruited using a convenience sampling method. Data on sociodemographic characteristics were collected, and assessments included the St. George’s Respiratory Questionnaire (SGRQ), spirometry, six-minute walk test (6MWT), and heart rate variability (HRV). Data were analyzed using standard statistical methods.

Results: A total of 47 patients were included in the study. Sociodemographic variables, habitual and environmental factors, spirometric characteristics, and clinicophysiological profiles were analyzed in relation to COPD parameters. No significant correlation was found between the body mass index, airflow obstruction, dyspnea, and exercise capacity (BODE) index and HRV parameters, nor between COPD severity and HRV frequency- and time-domain measures. However, a significant negative association was observed between the duration of COPD and HRV time-domain parameters. Specifically, COPD duration was negatively correlated with the root mean square of successive differences between normal heartbeats (rMSSD; r = -0.324, p = 0.026). Additionally, a significant negative correlation was found between the SGRQ impact score and standard deviation of NN intervals (SDNN).

Conclusion: These findings support the concept that autonomic dysregulation is an integral component of COPD pathophysiology. HRV is a simple, non-invasive biomarker for assessing autonomic nervous system function in patients with COPD and may help identify those at increased cardiovascular risk. Incorporating HRV evaluation, particularly through standardized long-term monitoring, into routine clinical assessment may enhance disease stratification, prognostication, and personalized management.

## Introduction

The Global Burden of Disease Study 2017 reported an increasing trend in the prevalence of non-communicable diseases (NCDs), including ischemic heart disease (IHD), chronic obstructive pulmonary disease (COPD), and stroke. The World Health Organization (WHO) Global Action Plan for the Prevention and Control of NCDs, aligned with the United Nations 2030 Agenda for Sustainable Development, highlights that COPD accounted for approximately 81.6 million disability-adjusted life years (DALYs) and 3.2 million deaths in 2017, with projections suggesting it may become the fourth leading cause of death by 2040 [1]. Among NCDs, COPD remains a major concern due to its high morbidity and increasing prevalence, driven by both pulmonary and systemic manifestations. Airflow obstruction in COPD significantly impairs gas exchange and cardiac function, leading to widespread systemic effects. Furthermore, the inflammatory milieu in COPD promotes a “spillover” of cytokines into the systemic circulation, exacerbating comorbid conditions such as IHD, heart failure, osteoporosis, sarcopenia, cachexia, normocytic anemia, lung cancer, depression, and diabetes [2].

Low- and middle-income countries account for approximately 90% of COPD-related deaths, with socioeconomic factors contributing significantly to this disproportionate burden.

Halbert et al. [3], in a review of the global burden of COPD, reported a pooled prevalence of 8.9% based on spirometric estimates, with physiologically defined COPD affecting approximately 9%-10% of adults aged ≥40 years [3].

Abnormal autonomic regulation of the cardiopulmonary system is a potential pathophysiological mechanism in COPD. Airway tone is regulated by parasympathetic cholinergic and sympathetic adrenergic pathways, as well as humoral factors [4,5]. Variations in oxygenation and the use of sympathomimetic agents further influence sympathetic nervous system activity. Patients with COPD often experience hypercapnia, hypoxemia, and fluctuations in intrathoracic pressure, all of which can affect autonomic balance. Experimental evidence suggests that autonomic dysfunction, characterized by increased sympathetic activity, may further amplify inflammatory responses [6]. Additionally, studies have demonstrated a link between oxidative stress in chronic lung disease and heightened sympathetic activity, suggesting a role in dysautonomic modulation [4].

Several studies have demonstrated that patients with COPD exhibit significant alterations in autonomic function [7]. COPD frequently coexists with cardiovascular disease, contributing to increased morbidity and mortality. Over the past two decades, growing attention has been directed toward the relationship between autonomic dysfunction and cardiovascular mortality [8]. Early detection of autonomic impairment in COPD patients may help identify those at higher risk who require closer monitoring. Heart rate variability (HRV) is a well-established, noninvasive, and cost-effective tool for assessing autonomic nervous system function. Its role in detecting early cardiovascular dysfunction is well recognized [9]. Within physiological limits, higher HRV is generally associated with better health status, whereas reduced HRV has been linked to an increased risk of cardiovascular disease [10].

COPD is also associated with structural and functional alterations in multiple organ systems, including malnutrition, weight loss, muscle weakness, and psychological comorbidities such as anxiety and depression. However, limited studies have explored the relationship between autonomic dysfunction and clinical severity, quality of life, and psychological well-being in patients with stable COPD. Despite increasing recognition of autonomic dysregulation in COPD, the relationship between HRV and disease severity, duration, and patient-reported outcomes remains incompletely understood. With this background, the present study aimed to assess autonomic dysfunction in patients with stable COPD using HRV. The primary objective was to evaluate the association between HRV and clinical characteristics of COPD, including the body mass index, airflow obstruction, dyspnea, and exercise capacity (BODE) index and disease severity based on GOLD classification. Secondary objectives included assessing the relationship between HRV and disease duration, symptom burden, and psychological status, including anxiety and depression. HRV was evaluated alongside established clinical tools, including the St. George’s Respiratory Questionnaire (SGRQ) [11], BODE index [12], and the Hospital Anxiety and Depression Scale (HADS) [13].

This study was designed as an exploratory observational analysis. The findings were previously presented as a meeting abstract at the 2025 American Thoracic Society International Conference on May 18, 2025 [14].

## Materials and methods

In this prospective observational study, patients with stable COPD who met the inclusion and exclusion criteria were recruited after obtaining informed consent. Patients aged ≥40 years with a physician diagnosis of COPD and a smoking history of more than 10 pack-years were included. Exclusion criteria comprised a history of asthma, pneumonia, or active tuberculosis, chronic respiratory failure, coronary artery disease, congestive heart failure, chronic kidney disease, obstructive sleep apnea, malignancy, coexisting type 2 diabetes mellitus, electrolyte disturbances, psychological comorbidities, and any acute exacerbation of COPD within the preceding two weeks. The sample size was determined based on patient availability within the study setting. A total of 47 patients were enrolled over a 12-month period. The study protocol was reviewed and approved by the Institutional Human Ethics Committee of PSG Institute of Medical Sciences and Research (Project No. 19/315).

Patient history and sociodemographic data were collected using a structured interview. Patients were classified according to the Global Initiative for Chronic Obstructive Lung Disease (GOLD) staging system (mild, moderate, severe, and very severe) based on post-bronchodilator Forced expiratory volume in one second (FEV₁) values. Additional clinical stratification included the modified Medical Research Council (mMRC) dyspnea scale [15], number of exacerbations in the previous year, and number of hospitalizations in the previous year. Validated instruments, including the SGRQ [11] and the HADS [13], were administered. Spirometry was performed to assess both pre- and post-bronchodilator responses. All patients underwent assessment using the BODE index [12] and the six-minute walk test (6MWT) [16], with the distance covered recorded. HRV was assessed using a dedicated HRV software. Autonomic cardiac activity was measured using a five-minute ambulatory ECG recording (NIVIQURE digital system) in lead II. Both time-domain and frequency-domain HRV parameters were analyzed. Data preprocessing and artifact correction were performed using built-in software algorithms. Given the exploratory nature of the study, adjustments for multiple comparisons were not performed. Potential confounders, such as medication use, were recorded but not included in multivariable analysis due to the limited sample size.

Data collected in the case record proforma were coded and entered into a master database for analysis. Copyright permissions were obtained for the use of the SGRQ and HADS instruments. The 6MWT was conducted in accordance with American Thoracic Society (ATS) guidelines.

Statistical analysis was performed using IBM SPSS Statistics for Windows, Version 16.0 (Released 2007; IBM Corp., Armonk, NY, USA). Continuous variables were expressed as mean ± standard deviation (SD) and compared using Student’s t-test for normally distributed data or appropriate nonparametric tests for skewed data. Categorical variables were expressed as proportions and compared using the chi-square test. Analysis of variance (ANOVA) with Bonferroni correction was used for comparisons involving three or more groups. Pearson’s correlation coefficient was used to assess relationships between continuous variables. A p-value < 0.05 was considered statistically significant.

## Results

A total of 47 patients were recruited for the study. The mean age of the participants was 62.98 ± 6.63 years, comprising 42 males (89.4%) and 5 females (10.6%). As overcrowding and both indoor and outdoor air pollution are recognized risk factors for COPD, the residential distribution of participants was assessed. Twenty patients (42.6%) resided in urban areas, while 27 (57.4%) resided in rural areas. Occupational history was also emphasized, as exposure to occupational hazards is an important contributor to airway obstruction in COPD. Eight patients (17.0%) were employed in industries with a history of dust exposure, 13 (27.7%) were engaged in agriculture, and 9 (19.1%) worked in the textile sector. One patient (2.1%) was unemployed, and another one (2.1%) was employed in a professional occupation. The remaining 15 patients (31.9%) were engaged in low-risk occupations without significant exposure, primarily daily wage labor. The sociodemographic characteristics are summarized in Table 1.

**Table 1:** Sociodemographic characteristics of the study population (N = 47)

Characteristics	Groups	Values
Age (in years)		63.98 ± 6.63
Gender	Male	42 (89.4%)
	Female	5 (10.6%)
BMI (kg/m^2^)		21.8 ± 3.7
Dwelling	Urban	20 (42.6%)
	Rural	27 (57.4%)
Education	Illiterate	12 (25.5%)
	Read/write	9 (19.1%)
	Primary	16 (34.0%)
	Higher secondary	5 (10.6%)
	Diploma	3 (6.4%)
	Graduate	2 (4.3%)
Socioeconomic status	Class I	33 (70.2%)
	Class II	13 (27.7%)
	Class III	0 (0%)
	Class IV	1 (2.1%)
	Class V	0 (0%)
Occupation	Unemployed	1 (2.1%)
	Agriculture	13 (27.7%)
	Textiles	9 (19.1%)
	Industry	8 (17%)
	Professional	1 (2.1%)
	Others	15 (31.9%)

The smoking status of the study participants was evaluated. Thirty-four patients (72.3%) were ex-smokers, eight (17.0%) were current smokers, and five (10.6%) were non-smokers. The mean smoking exposure was 23.4 ± 16.1 pack-years. Regarding alcohol consumption, 22 patients (46.8%) were non-alcoholic, 21 (44.7%) had a past history of alcohol intake, and 4 (8.5%) were current alcohol consumers. With respect to environmental exposures such as dust, industrial smoke, biomass fuel, environmental tobacco smoke, and pet animals, 14 patients (29.8%) reported no history of exposure. In contrast, 24 (51.1%) had multiple exposures, including occupational dust and smoke exposure; 3 (6.4%) reported dust exposure only; 4 (8.5%) reported biomass exposure; and 1 patient (2.1%) reported exposure to environmental tobacco smoke and industrial smoke, respectively. No participants reported exposure to pet animals.

Since the presence of comorbid illnesses influences outcomes in patients with COPD, we evaluated comorbidity profiles in the study population. Systemic hypertension was the most common comorbidity, observed in 17 patients (36.2%), followed by type 2 diabetes mellitus in 7 patients (14.9%). A history of pulmonary tuberculosis was present in 7 patients (14.9%), stroke in 2 patients (4.3%), and hypothyroidism in 1 patient (2.1%). Coronary artery disease, chronic kidney disease, and obstructive sleep apnea were not reported in any of the study participants. The comorbidity profile is summarized in Table 2. Regarding the background characteristics of COPD, the mean duration of illness was 9.72 ± 6.9 years, with a range of 1-30 years. Twenty-two patients (46.8%) had experienced at least one exacerbation in the previous year, while 25 (53.2%) had no exacerbations. Among those with exacerbations, 9 patients (19.1%) had ≥1 hospitalization in the past year, and 2 patients (4.3%) required ventilatory support. In terms of treatment characteristics, 25 patients (53.2%) were on triple inhaler therapy, including long-acting beta-agonists (LABA), inhaled corticosteroids, and long-acting muscarinic antagonists (LAMA). Seventeen patients (36.2%) received a combination of inhaled corticosteroids and LABA, while 2 patients (4.3%) were treated with a combination of LAMA and LABA. Three patients (6.4%) had no prior history of inhaled bronchodilator use. Table 2 summarizes the background COPD profile of the study population.

**Table 2:** Personal, comorbidity profile, and background characteristics of COPD of the study participants (N = 47) CVA: cerebrovascular accident, COPD: chronic obstructive pulmonary disease, NIV: non-invasive ventilation, IMV: invasive mechanical ventilation, ICS: inhaled corticosteroid, LABA: long-acting beta-agonist, LAMA: long-acting muscarinic antagonist.

	Groups	Values
Personal characteristics		
Smoking status	Current smoker	8 (17%)
	Ex-smoker	34 (72.3%)
	Never smoked	5 (10.6%)
Smoking pack years	Smoking pack years, mean ± SD	23.4 ± 16.1
	Current alcohol intake, n (%)	4 (8.5%)
	Past history of alcohol intake, n (%)	21 (44.7%)
	Non-alcoholic, n (%)	22 (46.8%)
Exposure to environmental hazards	No exposure, n (%)	14 (29.8%)
	Biomass, n (%)	4 (8.5%)
	Dust, n (%)	3 (6.4%)
	Industrial smoke, n (%)	1 (2.1%)
	Environmental tobacco smoke, n (%)	1 (2.1%)
	Multiple exposure source, n (%)	24 (51.1%)
	Pet animals, n (%)	0 (0%)
Comorbidity profile		
Comorbidity profile	Diabetes mellitus, n (%)	7 (14.9%)
	Systemic hypertension, n (%)	17 (36.2%)
	Hypothyroidism, n (%)	1 (2.1%)
	Coronary artery disease, n (%)	0 (0%)
	Chronic kidney disease, n (%)	0 (0%)
	Old CVA, n (%)	2 (4.3%)
	Old pulmonary tuberculosis, n (%)	7 (14.9%)
Background characteristics of COPD		
Duration of illness (in years)	Mean ± SD	9.72±6.9
Exacerbation in the last one year	No exacerbation, n (%)	25 (53.2%)
	At least one exacerbation, n (%)	22 (46.8%)
Hospitalization for acute exacerbation in the last one year	No hospitalization, n (%)	38 (80.9%)
	≥1 hospitalization, n (%)	9 (19.1%)
Ventilatory support (NIV/IMV) in the last one year	No support, n (%)	45 (95.7%)
	≥1 ventilatory support, n (%)	2 (4.3%)
Treatment taken (inhaler)	Not on treatment, n (%)	3 (6.4%)
	ICS + LABA, n (%)	17 (36.2%)
	ICS + LABA + LAMA, n (%)	25 (53.2%)
	LABA + LAMA, n (%)	2 (4.3%)

Baseline spirometric characteristics were assessed in all 47 patients. The mean post-bronchodilator FEV₁/FVC ratio was 61.9 ± 9.2%, consistent with airway obstruction according to GOLD criteria. The mean post-bronchodilator FEV₁% predicted was 45.5 ± 18.5%. Based on the GOLD classification of COPD severity, 16 patients (34.0%) had moderate disease (FEV₁ 50%-80% predicted), 21 patients (44.7%) had severe disease (FEV₁ 30%-50% predicted), and 6 patients (12.8%) had very severe disease (FEV₁ <30% predicted). The spirometric characteristics are summarized in Table 3. The mean room air oxygen saturation in the study population was 96.2 ± 1.6%, with a range of 90%-99%. The mMRC dyspnea grading showed that 18 patients (38.3%) were in Grade I, 21 (44.7%) in Grade II, and 7 (14.9%) in Grade III. The mean distance covered in the 6MWT was 369.36 ± 95.5 m (Table 3).

**Table 3:** Spirometric evaluations and clinical parameters of study population (N = 47) FVC: forced vital capacity, FEV₁: forced expiratory volume in one second, COPD: chronic obstructive pulmonary disease, mMRC: modified Medical Research Council, 6MWT: six-minute walk test, GOLD: Global Initiative for Chronic Obstructive Lung Disease.

Parameter	Value (mean ± SD or n (%))
Pre-bronchodilator FVC (mL)	1.61 ± 0.73
Pre-bronchodilator FVC (% predicted)	53.5 ± 20.9
Pre-bronchodilator FEV₁ (mL)	1.03 ± 0.52
Pre-bronchodilator FEV₁ (% predicted)	42.0 ± 18.83
Pre-bronchodilator FEV₁/FVC (%)	61.1 ± 8.1
Post-bronchodilator FVC (mL)	1.72 ± 0.73
Post-bronchodilator FVC (% predicted)	57.13 ± 19.96
Post-bronchodilator FEV₁ (mL)	1.10 ± 0.53
Post-bronchodilator FEV₁ (% predicted)	45.5 ± 18.5
Post-bronchodilator FEV₁/FVC (%)	61.9 ± 9.2
COPD severity (GOLD 2022)	
Mild	4 (8.5%)
Moderate	16 (34.0%)
Severe	21 (44.7%)
Very severe	6 (12.8%)
Heart rate (beats/min)	87.5 ± 11.9
Systolic BP (mmHg)	127.02 ± 13.5
Diastolic BP (mmHg)	77.0 ± 8.8
Respiratory rate (/min)	17.0 ± 1.0
Oxygen saturation (%)	96.2 ± 1.6
mMRC dyspnea grading	
Grade 0	1 (2.1%)
Grade 1	18 (35.3%)
Grade 2	21 (44.7%)
Grade 3	7 (14.9%)
Grade 4	0 (0%)
6MWT (meters)	369.36 ± 95.5
Anxiety	6 (12.7%)
Depression	4 (8.5%)

COPD phenotyping among the study population showed 17 patients (36.2%) in Group A, 23 (48.9%) in Group B, 2 (4.3%) in Group C, and 5 (10.6%) in Group D. The mean SGRQ scores were 39.16 ± 18.36 for the total score, 59.1 ± 21.4 for the activity component, 38.92 ± 18.14 for symptoms, and 27.6 ± 19.2 for impact. The BODE index showed that 16 patients (34.0%) had scores of 0-2, 18 (38.2%) had scores of 3-4, 7 (14.8%) had scores of 5-6, and 6 (12.7%) had scores of 7-10 (Table 4).

**Table 4:** Heart rate variability parameters according to the BODE index, COPD severity (PFT), and COPD phenotype (GOLD classification) BODE: BMI, airflow obstruction, dyspnea, exercise capacity, HRV: heart rate variability, HF: high frequency, LF: low frequency, SDNN: standard deviation of NN interval, HR: heart rate, pNN50: percentage of adjacent NN intervals that differ from each other by more than 50 ms, rMSSD: root mean square of successive differences between normal heartbeats.

Heart rate variability						
According to the BODE index	Variables	0-2 points	3-4 points	5-6 points	7-10 points	p-value
	HF	84.5 ± 7.7	82.3 ±9.3	84.7 ± 9.3	82.5 ± 7.4	0.836
	LF	15.4 ± 7.7	17.6 ±8.8	15.1 ± 9.4	17.4 ± 7.4	0.835
	HF/LF	8.3 ± 7.1	6.6 ± 5.0	8.3 ± 5.4	5.3 ± 1.9	0.634
	LF/HF	0.18 ± 0.11	0.22 ± 0.14	0.18 ± .14	0.21 ± .12	0.822
	SDNN	48.8 ±9.8	53.7 ± 11.4	44.8 ± 7.5	45.3 ± 9.9	0.147
	Average HR	84.8 ± 13.2	80.8 ± 13.7	89.7 ± 3.5	91.6 ± 0.4	0.336
	pNN50	0.49 ± 0.86	0.95 ± 1.0	0.21 ± .47	0.38 ± .94	0.232
	rMSSD	12.9 ± 6.6	18.6 ± 10.7	10.6 ± 5.1	11.6 ± 5.6	0.079
According to COPD severity (PFT)	Variables	Mild	Moderate	Severe	Very severe	p-value
	HF	81.9 ± 3.8	85.1 ± 9.8	81.9 ± 8.2	85.4 ± 4.6	0.604
	LF	18.0 ± 3.8	14.8 ± 9.8	18.0 ± 8.2	14.5 ± 4.6	0.607
	HF/LF	4.7 ± 1.3	9.3 ± 7.2	6.4 ± 5.1	6.5 ± 2.6	0.319
	LF/HF	0.21 ± 0.05	0.18 ± 0.15	0.22 ± .12	0.1 7± .06	0.694
	SDNN	54.6 ± 6.4	47.8 ± 9.7	50.2 ± 2.1	49.2 ± 9.2	0.704
	Average HR	78.0 ± 9.5	85.3 ± 14.1	85.9 ± 5.9	84.4 ± 5.2	0.809
	pNN50	1.0 ± 1.3	0.42 ± 0.81	0.67 ± .94	0.61 ± .99	0.646
	rMSSD	17.2 ± 7.0	12.2 ± 6.3	15.9 ± 0.9	14.2 ± 5.3	0.554
According to COPD phenotype (GOLD)	Variables	Group A	Group B	Group C	Group D	p-value
	HF	85.1 ± 7.4	81.9 ± 8.0	87.2 ± 8.3	83.2 ± 2.1	0.617
	LF	14.8 ± 7.4	17.9 ± 8.0	12.6 ± 8.5	16.7 ± 2.1	0.615
	HF/LF	8.4 ± 6.9	5.9 ± 3.6	8.9 ± 6.4	9.2 ± 8.3	0.415
	LF/HF	0.17 ± 0.10	0.22 ± 0.13	0.14 ± .10	0.20 ± 0.18	0.6
	SDNN	53.3 ± 11.8	47.1 ± 8.6	56.6 ± 3.8	46.2 ± 3.1	0.19
	Average HR	80.0 ± 14.2	88.4 ± 12.3	70.5 ± 4.3	90.7 ± 1.6	0.108
	pNN50	0.87 ± 1.05	0.41 ± 0.78	1.6 ± 1.5	0.24 ± .55	0.135
	rMSSD	16.0 ± 7.8	13.6 ± 9.9	20.3 ± 2.9	11.6 ± 5.1	0.546

**Figure 1:**
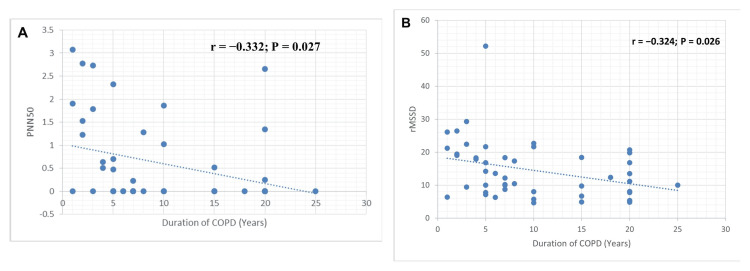
Correlation between duration of COPD and pNN50 and rMSSD Scatter plots depicting the relationship between disease duration (years) with (A) pNN50, an index of parasympathetic activity, and (B) rMSSD, a time-domain index of parasympathetic activity. COPD: chronic obstructive pulmonary disease, PNN50: percentage of successive RR intervals that differ by more than 50 ms, rMSSD: root mean square of successive differences between normal heartbeats.

The correlation between the BODE index and HRV parameters was not statistically significant (Table 4). Similarly, no significant association was observed between COPD severity and HRV frequency- or time-domain measures (Table 4). In addition, COPD phenotypes based on ABCD classification were not significantly associated with HRV parameters (Table 4). Pearson correlation analysis revealed a statistically significant negative association between COPD duration and HRV time-domain parameters. A modest negative correlation was observed between COPD duration and PNN50 (r = -0.332, p = 0.027) (Figure 1A).

**Figure 2:**
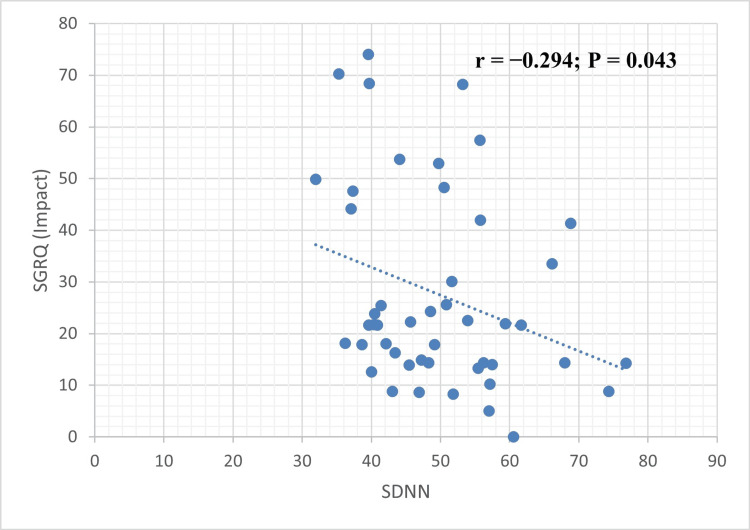
A statistically significant negative correlation was found between the SGRQ impact score and SDNN, another HRV time-domain measure (r = -0.294, p = 0.043) SGRQ: St. George’s Respiratory Questionnaire, SDNN: standard deviation of NN intervals, HRV: heart rate variability.

Similarly, COPD duration was negatively correlated with rMSSD (r = -0.324, p = 0.026) (Figure 1B). The corresponding coefficients of determination (R²) were 0.110 and 0.105, respectively, indicating that approximately 10%-11% of the variance in these HRV measures could be explained by COPD duration. In addition, a statistically significant negative correlation was observed between the SGRQ impact score and SDNN, another HRV time-domain parameter (r = -0.294, p = 0.043) (Figure 2). The coefficient of determination (R² = 0.086) suggested that approximately 8.6% of the variance in SDNN was explained by the SGRQ impact score.

**Figure 3:**
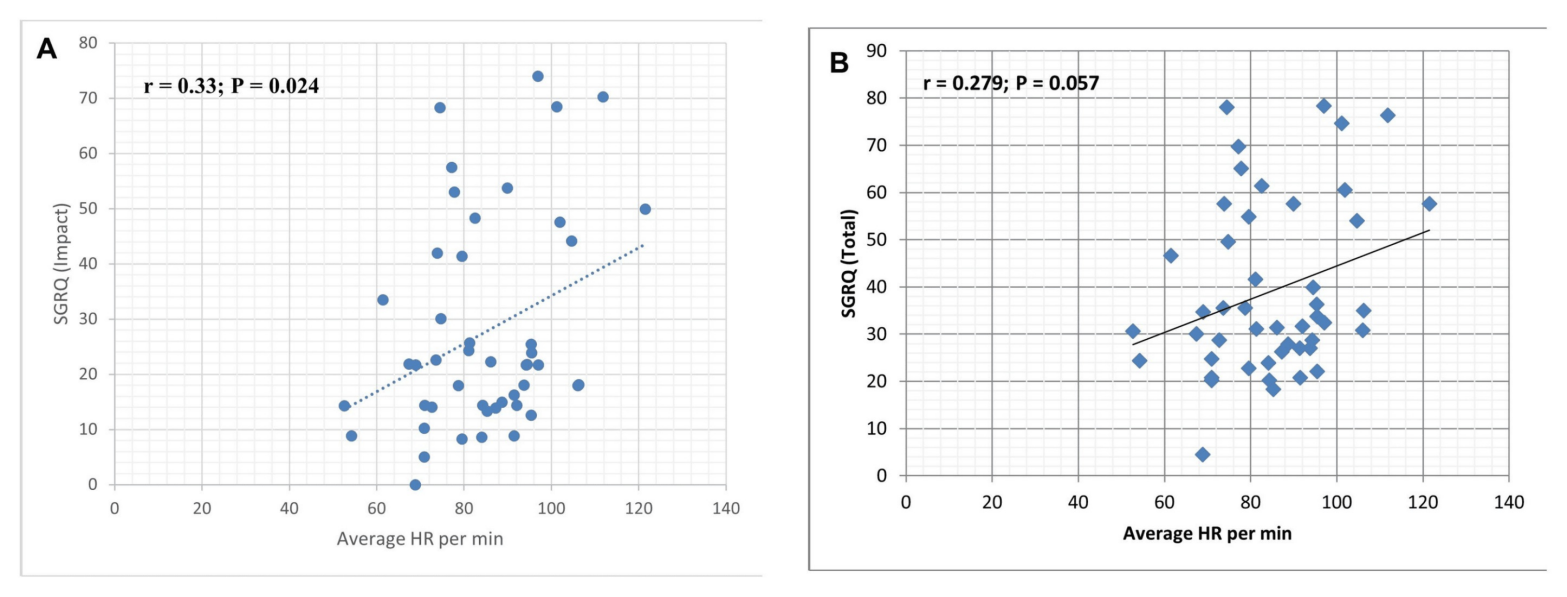
Correlation analysis of St. George’s Respiratory Questionnaire (SGRQ) impact score and total score with average heart rate Scatter plot demonstrating the relationship between (A) SGRQ impact score and average heart rate (beats per minute) and (B) SGRQ (total score) and average heart rate per minute.

In contrast to the negative associations observed between HRV parameters and disease severity, heart rate showed a modest positive correlation with both the SGRQ impact score (p = 0.024) and the total SGRQ score (p = 0.057) (Figures 3A, 3B).

This finding suggests that individuals with poorer health status or greater symptom burden tended to have higher resting heart rates, reflecting altered autonomic balance with reduced parasympathetic modulation.

**Figure 4:**
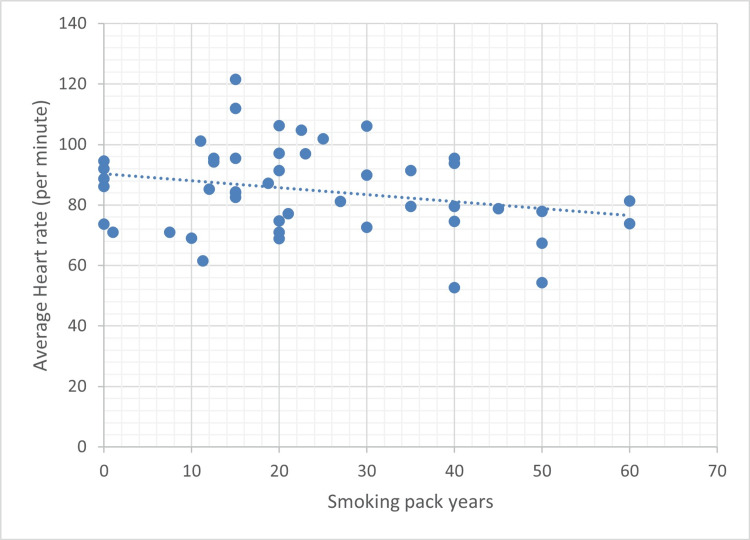
Correlation between smoking pack years and average heart rate Scatter plot showing the relationship between cumulative smoking exposure (pack years) and average heart rate (beats per minute). A weak negative correlation was observed (r = -0.253, p = 0.086), indicating a trend toward lower average heart rate with greater smoking exposure, though the association did not reach statistical significance.

Additionally, a moderate negative association was observed between average heart rate and smoking pack-years (p = 0.086), suggesting a potential inverse relationship that may warrant further investigation in larger cohorts (Figure 4).

## Discussion

Assessing HRV in the stable phase of COPD is particularly important, as it provides insight into baseline autonomic function independent of acute physiological stress. Although several studies have examined HRV in patients with COPD, most have focused on individuals during acute exacerbations, with relatively few including patients in the stable phase.

HRV has been extensively investigated as a marker of autonomic regulation in COPD and other chronic conditions. When interpreted in context, HRV offers a simple, inexpensive, and non-invasive tool for evaluating autonomic activity. Its potential role in predicting cardiovascular morbidity and mortality further enhances its clinical value in long-term disease management.

Health-related quality of life and disease severity in COPD are commonly assessed using validated instruments such as the SGRQ [11], the Chronic Respiratory Disease Questionnaire (CRQ), the London Chest Activity of Daily Living (LCADL) scale, and the COPD Assessment Test (CAT). Ferrer et al. demonstrated that, among patients with asthma and COPD, the predicted percentage of FEV₁ showed the strongest association with the SGRQ total score in a multiple linear regression model, underscoring the utility of the SGRQ as a measure of disease impact [17]. In the present study, the mean SGRQ total score among participants was 39.16 ± 18.36, indicating moderate impairment in health-related quality of life within the cohort.

Jensen et al. reported that resting heart rate increases with COPD severity (p < 0.001) and is independently associated with both cardiovascular and all-cause mortality across all stages of the disease [18]. In the present study, the mean resting heart rate was 87.5 ± 11.9 beats per minute, suggesting relative sympathetic dominance consistent with autonomic imbalance in COPD. We observed a statistically significant positive correlation between the SGRQ Impact score and average heart rate (r = 0.33, p = 0.024), and a positive trend between the SGRQ total score and average heart rate (r = 0.279, p = 0.057). The corresponding coefficients of determination (R² = 0.109 and 0.078, respectively) indicate that approximately 8%-11% of the variance in heart rate may be explained by these quality-of-life measures. Nevertheless, these findings require validation in larger controlled studies, as the explanatory power of our smaller sample is limited and should be interpreted with caution.

Previous studies have consistently demonstrated impaired cardiac autonomic modulation in patients with COPD. Zupanic et al. reported significant reductions in key time-domain and frequency-domain HRV parameters, including SDNN, rMSSD, pNN50, HF, and LF, among COPD patients compared with healthy controls [19]. SDNN, one of the most robust prognostic time-domain indices of HRV, also showed a significant negative correlation with the SGRQ Impact score in our study (r = -0.294, p = 0.043), reinforcing its value as an indicator of autonomic dysfunction and disease burden.

In this study, we observed modest but statistically significant associations between HRV parameters and disease duration, as well as quality-of-life measures. The strength of these correlations was limited, suggesting that HRV reflects only one component of the complex pathophysiology of stable COPD. Notably, no significant association was found between HRV parameters and conventional measures of disease severity, such as GOLD staging and the BODE index, in patients with stable COPD. This may indicate that autonomic dysfunction progresses independently of spirometric severity. An important consideration is that our study used short-term HRV measurements. Shaffer et al. [20] concluded that short-term HRV recordings have lower predictive value compared with long-term recordings, which remain the reference standard for predicting health outcomes. Another consideration is the difference in autonomic function between stable COPD and acute exacerbations. Zamarrón et al. [21] reported that patients with acute exacerbation of COPD (AECOPD) exhibit increased autonomic activity compared with those in the stable phase. In our study, the majority of participants (25, 53.2%) had no history of exacerbation in the past year. A systematic review and meta-analysis published in 2023 [22], which included 512 studies, reported that the relationship between COPD and HRV reduction remains unclear due to methodological heterogeneity and variability in autonomic modulation across different clinical contexts. Ganesan et al. found that time-domain HRV indices, such as mean heart rate and rMSSD, were significantly reduced (p < 0.005) in patients with moderate to very severe COPD compared with those with mild disease [23]. These findings suggest that cardiac autonomic dysregulation, characterized by reduced parasympathetic activity and increased sympathetic activity, worsens with disease severity. In line with these findings, our study demonstrated modest but significant negative correlations between COPD duration and HRV time-domain measures, specifically rMSSD (r = -0.324, p = 0.026) and pNN50 (r = -0.332, p = 0.027). These results suggest progressive impairment of parasympathetic function with longer disease duration.

Our findings are further supported by Chhabra et al., who reported that serum interleukin-6, an inflammatory marker elevated in COPD, inversely correlates with pNN50, highlighting the link between systemic inflammation and parasympathetic withdrawal [24]. Taken together, these data strengthen the evidence that reduced HRV in COPD reflects underlying autonomic imbalance associated with chronic inflammation and disease progression.

In the present cohort, more than half of the participants (53.2%) had no history of exacerbation in the preceding year, which may partially explain the absence of a significant association between HRV indices and COPD severity categories.

Additionally, our study used short-term HRV recordings, which, while practical and widely accepted for cross-sectional analysis, may have limited predictive accuracy compared with long-term assessments. Shaffer et al. (2020) emphasized that long-term HRV recordings represent the reference standard for evaluating autonomic function and predicting health outcomes, and that short- and long-term HRV measures should not be used interchangeably [20].

Despite these considerations, the consistent inverse relationships observed between COPD duration, symptom burden, and HRV parameters in our study provide further evidence of progressive autonomic dysregulation in COPD. These findings also highlight the potential of HRV as a non-invasive, cost-effective, complementary biomarker for assessing cardiovascular risk in this population. However, its role in clinical risk stratification remains to be clearly established in larger, controlled studies.

Limitations

This study has several limitations. (i) The small sample size (N = 47) and single-center design limit statistical power. (ii) The cross-sectional, exploratory, and observational nature of the study precludes causal inference between autonomic dysfunction and COPD severity, duration, or cardiovascular risk. (iii) HRV assessment was based on short-term recordings rather than 24-hour monitoring, which may have reduced sensitivity in detecting subtle autonomic alterations. (iv) Potential confounders, including medication use (e.g., beta-agonists), smoking status, and unrecognized cardiovascular comorbidities, were recorded but not fully adjusted for through multivariable analysis due to the small sample size. (v) The predominance of male participants limits generalizability. (vi) The absence of a healthy control group further limits extrapolation of the findings.

## Conclusions

Patients with stable COPD in this study demonstrated modest associations between autonomic imbalance, disease duration, and quality of life. Inverse correlations between HRV time-domain parameters (SDNN, rMSSD, and pNN50) and both disease duration and health-related quality of life (SGRQ scores) suggest reduced parasympathetic activity with advancing disease. In contrast, the positive association between resting heart rate and SGRQ scores indicates relative sympathetic predominance with greater symptom burden. These findings suggest that autonomic dysregulation may contribute to systemic cardiovascular involvement in COPD. However, given the exploratory nature of the study, the results should be interpreted with caution. HRV, as a non-invasive and accessible measure, may provide complementary insight into autonomic dysfunction in COPD alongside conventional clinical assessment, potentially helping to identify patients at increased cardiovascular risk and to support disease stratification and prognostication. Further large-scale, longitudinal, and controlled studies are needed to assess the prognostic utility of HRV in predicting morbidity and mortality in COPD.
